# FEASIBILITY AND PRELIMINARY EFFECTIVENESS OF IMPLEMENTATION OF THE PAIN-CPG-EIT

**DOI:** 10.1093/geroni/igae098.1807

**Published:** 2024-12-31

**Authors:** Rachel McPherson

**Affiliations:** University of Maryland, Baltimore, Maryland, United States

## Abstract

The primary aim of this pilot study, testing the implementation of the Pain-CPG-EIT, was to evaluate feasibility (delivery and enactment) and preliminary effectiveness. Delivery was based on the intervention being provided as intended and enactment was based on expected behaviors being performed. It was hypothesized that: (1) all components of the intervention would be delivered; and (2) the goals established by each community would be achieved as expected or greater than expected, there would be an increase in nonpharmacologic interventions, and a decrease in unnecessary pharmacologic medications. Effectiveness was based on evidence that residents would have a: (1) pain assessment completed, diagnosis for the pain, and an individualized pain management care plan developed; (2) decrease in pain; and (3) evaluation of appropriate use of opioids. This was a pragmatic trial and no identifiable data was obtained on residents. Participants had a mean age of 70 (SD=8), over half were male (N=25, 51%), and the majority were Black residents (N=33, 67%), Non-Hispanic (N=48, 98%), not married (N=45,92%) and had at least a high school education (N=37, 76%). The mean score on the BIMS was 6 (SD=4) they had 4 (SD=1) comorbidities. The intervention components were delivered as intended and goals achieved. There was significant improvement in the appropriate pain assessments, no change in diagnosis of pain, an increase in the percentage of residents with pain addressed in their care plan, some improvement in assessment for appropriate opioid use, and a decrease in the percentage of residents on opioids.

